# Rethinking statistical learning as a continuous dynamic stochastic process, from the motor systems perspective

**DOI:** 10.3389/fnins.2022.1033776

**Published:** 2022-11-08

**Authors:** Anna Vaskevich, Elizabeth B. Torres

**Affiliations:** ^1^Sensory Motor Integration Lab, Department of Psychology, Rutgers, The State University of New Jersey, New Brunswick, NJ, United States; ^2^Rutgers Center for Cognitive Science, Piscataway, NJ, United States; ^3^Rutgers Computer Science Department, Computational Biomedicine Imaging and Modeling Center, Piscataway, NJ, United States

**Keywords:** statistical learning, dynamic learning, exploration, stochastic process, error correction, active inference learning, reinforcement learning

## Abstract

The brain integrates streams of sensory input and builds accurate predictions, while arriving at stable percepts under disparate time scales. This stochastic process bears different unfolding dynamics for different people, yet statistical learning (SL) currently averages out, as noise, individual fluctuations in data streams registered from the brain as the person learns. We here adopt a new analytical approach that instead of averaging out fluctuations in continuous electroencephalographic (EEG)-based data streams, takes these gross data as the important signals. Our new approach reassesses how individuals dynamically learn predictive information in stable and unstable environments. We find neural correlates for two types of learners in a visuomotor task: narrow-variance learners, who retain explicit knowledge of the regularity embedded in the stimuli. They seem to use an *error-correction* strategy steadily present in both stable and unstable environments. This strategy can be captured by current optimization-based computational frameworks. In contrast, broad-variance learners emerge only in the unstable environment. Local analyses of the moment-by-moment fluctuations, naïve to the overall outcome, reveal an initial period of memoryless learning, well characterized by a *continuous* gamma process starting out exponentially distributed whereby all future events are equally probable, with high signal (mean) to noise (variance) ratio. The empirically derived continuous Gamma process smoothly converges to predictive Gaussian signatures comparable to those observed for the error-corrective mode that is captured by current optimization-driven computational models. We coin this initially seemingly purposeless stage *exploratory*. Globally, we examine a posteriori the fluctuations in distributions’ shapes over the empirically estimated stochastic signatures. We then confirm that the exploratory mode of those learners, free of expectation, random and memoryless, but with high signal, precedes the acquisition of the error-correction mode boasting smooth transition from exponential to symmetric distributions’ shapes. This early naïve phase of the learning process has been overlooked by current models driven by expected, predictive information and error-based learning. Our work demonstrates that (statistical) learning is a highly dynamic and stochastic process, unfolding at different time scales, and evolving distinct learning strategies on demand.

## Introduction

At the start of life, human babies gradually become aware of their bodies in motion and as they understand it, they come to own the consequences of impending movements that make up all their purposeful actions. Seemingly *purposelessly*, neonates explore their surroundings as they expand their limbs with antigravity motions and eventually learn to reach out to their immediate space in a well-controlled, *purposeful*, and intended manner. The type of highly dynamic, spontaneous, exploratory learning that is at first driven by surprise and curiosity, has no initial goal or desired target. At this early stage of learning, all future events are equally probable to the cognitive system. The learning is merely a wondering process, “*what happens if I do this?*”, perhaps a guess, “*if I do this, then this (consequence) will ensue, otherwise, this other (consequence) will happen*…”. The current work offers evidence to suggest that this endogenous and dynamic type of learning in early life may scaffold how we learn in general. That is, that before realizing that certain regularities are present in the environment we collect information spontaneously, without relying on prior knowledge, committing to some stimuli salient feature, or using referencing goals. This stage, that has so far been overlooked, is not well described by traditional models of error correction learning. These models rely on expectation and surprise minimization. However, there are situations whereby the system does not yet have referencing information to generate a prediction error or expected prediction error code.

Research about learning, whether in the perceptual, the motor, or the cognitive domain, is primarily based on error-correction schemas (Censor et al., 2012; Hasson, 2017; Frost et al., 2019). These schemas are aimed at reducing the difference between a desired configuration or goal to be learned, and the current learning state (Hasson, 2017). Such goals tend to be exogenous in nature, but implicit in them are rules that the system must find. Somehow the spontaneous self-discovery process that we relied on as babies, to learn about sensing our body in the world and sensing the world in our body, tends to fade away from our behavioral research. Indeed, curious exploration seldom enters our experimental paradigms in explicit ways (Frost et al., 2019). Some animal models of exploratory behavior (Drai and Golani, 2001) have nevertheless been successfully extended to characterize exploration in human infants as excursions that separate segments of movements’ development from lingering episodes (Frostig et al., 2020). This recent research suggests behavioral homology across species and prompted us to hypothesize that at a finer temporal learning scale, a wondering, exploratory code may hide embedded in the fluctuations of our performance. We tend to average out such fluctuations as superfluous noise, often referred to as gross data. Certainly, when favoring *a priori* imposed theoretical means under assumptions of normality and stationarity in the data registered during the learning process, we miss the opportunity to know what possible information lies in the gross data.

The exploratory code discussed above is not to be confused with the exploration mode that is commonly addressed in models of exploration *vs*. exploitation in reinforcement learning (RL) (Sutton, 1992; Dayan and Balleine, 2002). Within this computational framework, learning depends on a reward, which is either intrinsically obtained, or extrinsically provided. However, for both exploration and exploitation, the learning is best described by error correction, as the system considers information and aims to descent optimally along the gradient of some implicit objective function, minimizing the error towards a desirable configuration. The RL framework does not explain how the objective (target) of the objective function is determined neither does it say how the value of the target self-emerges in different contexts. This includes more recent work on intrinsically motivated RL, where “Curiosity thus seems to be a matter of finding the right balance so that the agent is constantly maximizing the rate of reducing the prediction errors” (Dubey and Griffiths, 2020). Indeed, RL solves a different problem than that of self-discovering the perceptual goal or objective of a given situation.

We here focus precisely on how the system comes to self-discover the task-goal or purpose by firstly opening information channels welcoming surprise. More specifically, we isolate the spontaneous exploratory mode of learning. This mode without expectations, or referencing signals, leads to the self-discovery of the goal or objective. To that end, we focus on the cognitive processes known as implicit or statistical learning (SL). While we recognize other influential computational frameworks such as active inference and Bayesian RL contribute to our understanding of learning in general (Friston et al., 2016, 2017), SL is ideal for the present study as it involves embedding and manipulating the predictability of specific regularities within the perceptual input, so that the emergence of expectations and transitions between different learning modes can be tracked online. We return to the relevance and implications of our results on other computational frameworks that rely on optimization and error-correction in the “Discussion” section.

Implicit SL describes the ability of the brain to extract (largely beneath awareness) regularities from the environment (Hasson, 2017; Frost et al., 2019; Conway, 2020). Such capacity has long been known to support a wide range of basic human skills such as discrimination, categorization, and segmentation of continuous information (Saffran et al., 1996; Romberg and Saffran, 2010; Christiansen, 2019) and predictive aspects of social interactions (Torres et al., 2013a; Sinha, 2014; Crivello et al., 2018). Previous research has consistently shown that regardless of the nature of the embedded regularity (motor, perceptual or both), SL involves motor control systems, so that when participants are required to respond, the presence of predictive information modulates both response preparation and response execution processes (Kunar et al., 2007; Schwarb and Schumacher, 2012; Vaskevich et al., 2021). Yet work to addresses the stochastic motor signatures of SL during motor decisions communicating a preferred stimulus is sparse (Torres et al., 2013a), particularly those involving different levels of neuromotor control (Torres, 2011).

In this work, we reevaluate SL from the standpoint of sensory-motor systems. We reasoned that the motor percept that emerges from the sensations of our own endogenously generated biorhythmic motions could serve to support the type of SL that other perceptual systems would experience to gain behavioral control. More specifically, we propose to reframe the SL problem using recent advances in developmental research of neuromotor control (Torres et al., 2016) that focuses on time series of biorhythmic signals like those derived from electroencephalographic (EEG) signals (Ryu et al., 2021). We track the dynamic changes in stochastic signature of the learning process, continuously evaluating an EEG signal recorded while participants perform in a learning task that contained predictive information (i.e., regularities).

**FIGURE 1:**
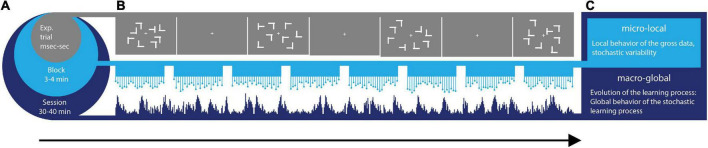
Dynamic statistical learning. **(A)** Different time scales of learning are accompanied by different types of learning supporting each level. From a level at sub-second time scales, to the scale of 40 min, different levels of granularity in the data afford different levels of precision to describe learning phenomena. Averaging out fluctuations in the system’s responses may eliminate gross data containing important information on learning mechanisms. These may be varying from trial to trial and from block to block at each level. **(B)** Visual search task: the target was a letter T rotated either left or right that appeared among rotated Ls (distractors). Across trials, the spatial configurations of target and distractors (i.e., layouts) could repeat (correlated group), be generated randomly (random group) or repeat on half of the trials (mixed group). **(C)** Micro-Local vs. Macro-Global signatures of variability are extracted from fluctuations in EEG signals recorded while participants searched for the target and pressed the corresponding response key as fast as possible.

To uncover the continuous dynamics of SL, we consider multiple time scales (Figure 1A) within the context of a visual search task (Figure 1B) whereby learning takes place across millisecond, minutes, and hours. Furthermore, we view the stochastic phenomena at a local and at a global level (Figure 1C). At the local level, we start naïve, without empirical knowledge of the stochastic process at hand. We do not make theoretical assumptions about this process (e.g., that is Gaussian, stationary, linear, etc.). Instead, we obtain moment by moment, the stochastic signatures of data parameters (e.g., signals’ amplitude and timing) and track how they evolve over time, as the learning unfolds. At the global level, we then examine *a posteriori*, the fluctuations in those stochastic signatures that we empirically estimated, to gain insight into the overall dynamics of the SL process that took place. For example, we track the evolution of the empirically estimated probability distributions’ shapes.

We analyze fluctuations of a continuous EEG signal, recorded during the visual search task. While we leverage the precise time stamping of the events in the data acquisition system and the use of stable and unstable implicit-learning environments (Vaskevich et al., 2021), we empirically estimate anew, moment by moment, the probability distribution function (PDF) that best fits fluctuations in the data and obtain the continuous family of PDFs describing the overall learning process. We let these fluctuations that are often discarded as gross data, reveal the primordial way of curious, exploratory learning, preceding the self-discovery of regularities conducive of a goal and eventually defining the error in the error-correction mode. We reframe SL from the point of view of a developing, nascent motor system that spontaneously transitions from purposeless to purposeful behavior.

## Materials and methods

This study involving human participants was reviewed and approved by the Institutional Review Board of Tel Aviv University. The participants provided their written informed consent to participate in this study. Behavioral and ERP analyses of these data were previously published (Vaskevich et al., 2021). Here we focus on the continuous EEG signal, without taking data epochs and averaging data parameters under theoretical assumptions of normality, linearity, and stationarity. Instead, we empirically estimate the continuous family of PDFs that in a maximum likelihood (MLE) sense, best fits what is traditionally discarded as superfluous gross data. This novel approach enabled us to isolate phenomena that cannot be observed when data is analyzed with conventional methods, leading to the uncovering of entirely new results.

### Participants

Data from 70 participants (48 female, mean age, 23.7) was analyzed in this study: 24 in the random group, 23 in the correlated, and 23 in the mixed groups. There were no differences in age or gender between the three experimental groups. Two participants (one in the mixed group and one in the correlated group) were removed from the analyses due to incomplete data: their EEG recording started late, missing the first few trials. As we focus here on continuous data analyses of the full learning experience, these two subjects were excluded.

### Stimuli and procedure

All participants gave informed consent following the procedures of a protocol approved by the Ethics Committee at the Tel Aviv University. The EEG signal was recorded during the visual search task. This task was followed by an explicit memory test during which EEG was not recorded. A more detailed account of the procedure can be found in Vaskevich et al. (2021).

Stimuli in the visual search task and the explicit memory test were white T’s and L’s (Figure 1B). All stimuli were made up of two lines of equal length (forming either an L or a T). From a viewing distance of approximately 60 cm, each item in the display subtended 1.5° × 1.5° of visual angle. All items appeared within an imaginary rectangle (20° × 15°) on a gray background with a white fixation cross in the middle of the screen (0.4° × 0.4°). Targets appeared with equal probability on the right or left side of the screen.

#### Visual search task

Participants searched for a rotated T (target) among heterogeneously rotated L’s (distractors) while keeping their eyes on the fixation cross. Each trial began with the presentation of a fixation cross for 2,100, 2,200, or 2,300 ms (randomly jittered) followed by an array of one of two possible targets (left or right rotated T) among seven distractors. Participants were instructed to press a response key corresponding to the appropriate target as fast as possible -i.e., the goal of the task was to be accurate as fast as possible. Each participant was randomly assigned to one of three groups, with the degree of regularity in the task varying along a gradient. At one extreme the participants searched for the target within a highly predictable environment where predefined spatial configurations of target and distractors (layouts) were repeated from trial to trial (correlated group). Presumably, the embedded regularity can be easily and systematically confirmed by the system. At the other extreme, participants experienced the least amount of regularity, as from trial to trial, the layouts of the display were generated randomly (random Group). For the third group, consistent and random layouts were mixed throughout the task (mixed group). Any regularity cumulatively built from random guesses and confirmations, thus creating the ground for self-emergence of the overall goal or purpose of the task. This task is ideal to investigate the dynamic progression of SL. The gradient of predictability enables to examine, moment by moment, stochastic variations in learning between environments that differ in the reliability of predicting and confirming a guessed regularity. Depending on the group, the visual search contained the consistent mapping condition (correlated group), the random mapping condition (random group), or both (mixed group).

In summary, the three groups corresponded to predictable predictability (consistent group), predictable unpredictability (random group) and unpredictable predictability (mixed group). We were particularly interested in learning in the mixed group relative to the other two (predictable) groups.

For the consistent mapping condition, spatial configurations of targets and distractors were randomly generated for each participant (8 layouts for the mixed group and 16 layouts for the correlated group). In the random mapping condition targets and distractors appeared in random locations throughout the task. The order of layouts was randomized every 16 trials (in the case of the mixed group 16 trials correspond to eight consistent and eight random trials presented in a random order). The identity of the target (left or right rotation) was chosen randomly on each trial and did not correlate with the spatial regularity. Participants completed 512 trials in the experiment. Only correct trials were included in the analysis.

#### Explicit memory test

Participants were not informed of the regularity in the visual search task. Upon completing the task, participants in the mixed and correlated groups (when the task contained regularity) completed an explicit memory test, designed to reveal explicit knowledge of the regularity: participants saw the layouts that were presented to them during the search task mixed with new, randomly generated layouts. For each layout participants had to indicate whether they have seen the layout during the visual search task or not. We then computed an Explicit Memory Test (ET) score (hit rate/false alarm rate) that is considered to reflect each participant’s explicit knowledge of the regularity, so that higher scores correspond to better explicit knowledge (Vaskevich et al., 2021).

#### EEG recording

Electroencephalographic signals were recorded inside a shielded Faraday cage, with a Biosemi Active Two system (Biosemi B.V., Amsterdam, Netherlands), from 32 scalp electrodes at a subset of locations from the extended 10–20 system. The single-ended voltage was recorded between each electrode site and a common mode sense electrode (CMS/DRL). Data was digitized at 256 Hz (for a more detailed account see Vaskevich et al., 2021). We rely on continuous recordings, without averaging epochs of the data. In this work, we focus on the electrodes that do not reflect strong eye muscle activity either through blinking or the jaw movement. The analyzed subset Fp1, Fp2, AF3, AF4, F3, F4, F7, F8, Fz, FCz, C3, C4, Cz, T7, T8, P1, P2, P3, P4, P5, P6, P7, P8, Pz, PO3, PO4, PO7, PO8, POz, O1, O2, and Oz), includes all the electrodes that were previously analyzed (P7, P8, PO3, PO4, PO7, PO8, C3, C4). We use the EEGLAB PREP pipeline (Bigdely-Shamlo et al., 2015) to clean the EEG signals.

#### Cross-coherence analyses and network representation

The statistical analyses described in the next sections were done for a hub channel, chosen continuously for each time window (5 s of recording) with 50% overlap of the sliding window. Here we describe the process by which these hub channels were selected. Based on previous work with the same approach we bandpass filtered the data at 13–100 Hz using IIR filter at 20th order (Ryu et al., 2021). Two sample leads, taken pairwise across all sensors of the EEG cap were then used to instantiate the analyses. We used cross-coherence to quantify the similarity between any two leads (Phinyomark et al., 2012). For each pair, the maximal cross-coherence was obtained, with corresponding phase and frequency values at which the maximum was attained. The maximal cross-coherence matrix was used as an adjacency matrix to build a weighted undirected graph representation of a network (Supplementary Figure 1). Next, network connectivity analyses were used to obtain the maximum clustering coefficient representing the hub within each time window at the selected frequency band. The stochastic signatures of the moment-by-moment fluctuations in neural activity were then tracked in each overlapping window for the identified hub.

### New data type: The micro-movement spikes

**FIGURE 2:**
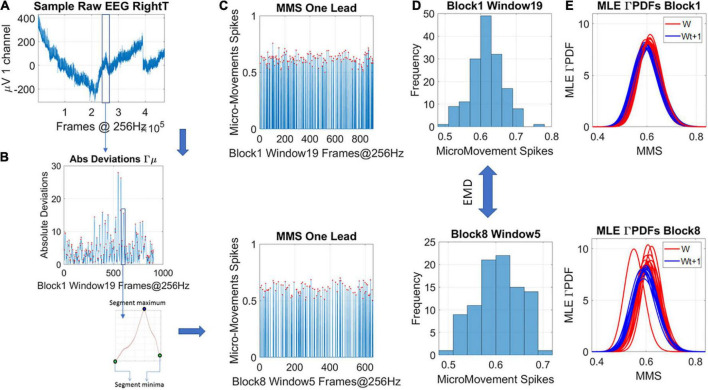
Transforming continuous analog signals to digital spikes: micro-movement spikes MMS. **(A)** Sample electroencephalographic signal from one hub channel determined through network connectivity analyses, zooming into one segment. Sweeping through the signal, windows of 5 s with 50% overlap are taken to scale each peak value deviated from the empirically estimated mean (μV). **(B)** For each participant, the original peaks are used to empirically estimate the mean amplitude across the session, and obtain, for each point in the time series, the absolute deviation from the mean. This series of fluctuations are then used to scale out possible allometric effects from e.g., anatomical head differences, using equation 1 in the methods. **(C)** The unitless, standardized MMS are plotted as time series conserving the original peaks’ timing, shown here for two sample states in some window of blocks 1 and 8. **(D)** The peaks (red dots) are gathered into a frequency histogram to obtain the histogram’s difference, from window to window (block by block), using the earth movers’ distance, a similarity metric used in transport problems. We then obtain the amount of effort that it takes to transform one frequency histogram into the other. **(E)** Using maximum likelihood estimation (MLE) the best continuous family of probability distributions fitting the frequency histogram is obtained, shown here for different time windows.

The analysis that is at the heart of the current work relies on the micro movements (MMS) spikes. This type of data and analytical platform, developed in the Torres lab (Torres et al., 2013a), and patented by the US Patent office (Torres, 2018a), was used in the current work to examine the change in stochastic variations of an EEG signal over time. To obtain the MMS of the EEG-hub biorhythmic signal, for each participant we take the peaks of the original EEG-hub waveform, derive the empirical distribution of the peaks and using the empirically estimated mean, we obtain the absolute deviation of each time point in the EEG-hub time series, from the empirically estimated mean. In the present data, the continuous Gamma family of probability distributions best fitted the peaks data, in an MLE sense. The Gamma family has well defined moments. We used the empirically estimated mean amplitude (μV) in our computations, to track the moment-by-moment fluctuations away from the empirically estimated mean. This builds a time series of micro-movements’ spikes (MMS) which consists of periods of activity away from the mean interspersed with quiet period of mean activity. Importantly, we retained the original times where those fluctuation peaks occurred and built normalized spike trains using the deviations from the mean amplitude using equation (1). An example is shown in Figures 2A,B.

Equation 1 scales out allometric effects owing to anatomical differences (Lleonart et al., 2000). Each local peak (max) of these series of fluctuations is divided by the sum of its value and the averaged values of points between the two local minima surrounding it


(1) $$M\,M\,S=\frac{\max}{\max+a\,v\,g_{\min-t\,o-\min}}$$

The result is then plotted, reflecting the unitless standardized MMS (Figure 2C), which describe the minute fluctuations in the original waveform (the EEG-hub), away from the empirically estimated mean (Figure 2C). Sweeping through the MMS trains, the values of the peaks (ranging now between 0 and 1) are gathered into frequency histograms for windows of 5 s with 50% overlap between each two consecutive windows (Figure 2D shows the corresponding histograms from the sampled blocks and windows in Figure 2C). We explored between 1- and 5-s-long windows (with 50% overlap) and settled on 5 s as the minimal time unit that gave us acceptable 95% confidence intervals in the empirical estimation process requiring 100 peaks or more.

### A similarity metric for abstract probability spaces

The Earth Mover’s Distance, EMD (Monge, 1781; Rubner et al., 1998) was used to obtain the scalar difference from moment to moment between the frequency histograms. This built a time series of such scalar quantity and enabled quantification of the dynamically changing stochastic trajectories. Figure 2D shows two sample histograms that can serve as input to the EMD metric expressing this (abstract) distance notion in probability space. Figure 2E shows an example of the empirically estimated Gamma PDFs across windows, contrasting blocks 1 and 8 for two quadrants of the Gamma parameter plane where these points are to be represented (see next section).

### Local analyses: Empirical estimation of gamma scale and shape parameters

Upon deriving the MMS, we proceed to sweep through them using 5-s-long windows of MMS activity, with 50% overlap. This gives us a local estimation (at each window) of the stochastic process. Using MLE, we empirically estimate the shape and scale of the best PDF in an MLE sense. Examples of frequency histograms are shown in Figure 2D for different sample blocks and windows. Examples of PDFs are shown in Figure 2E. We found that the continuous Gamma family of PDFs were the best MLE fit for these windows of normalized MMS activity. Among distributions that we tested were the Lognormal, the normal, the exponential, the Gamma and the Weibull.

**FIGURE 3:**
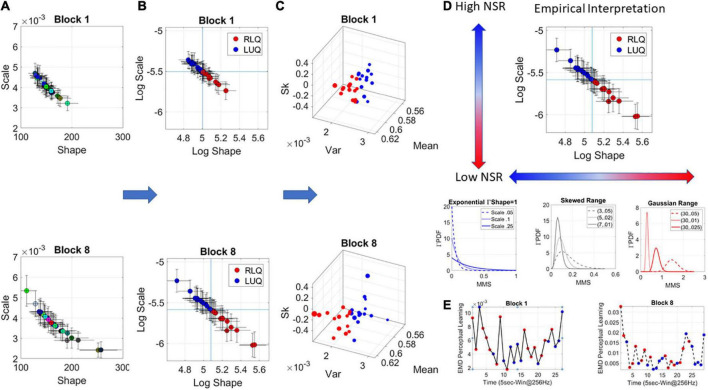
Stochastic analyses of the MMS derived from hub’s activities. **(A)** Upon determination of the lead the MMS are obtained and MLE used to determine the parameters of the best continuous family of probability distribution functions (PDFs) describing their fluctuations. In this case the Gamma family. The Gamma shape and scale parameters thus estimated, are then plotted with 95% confidence intervals, on the Gamma parameter plane. **(A)** Each point represents the signatures of a 5-s window with 50% overlap. Colors represent arbitrary order. **(B)** The log–log Gamma parameter plane is obtained to track points according to the quadrants spanned by the median shape and median scale, taken across each block. The Right Lower Quadrant (RLQ) contrasts with the Left Upper Quadrant (LUQ). **(C)** The Gamma moments are obtained to visualize the points in **(B)** on a parameter space whereby the Gamma mean is represented along *x-axis*, the variance along the *y-axis*, the skewness along the *z-axis* and the size of the marker is proportional to the kurtosis. The color corresponds to the direction of the shift, where the point lands, red is from the LUQ to the RLQ, or from the RLQ to itself, whereas blue is from the RLQ to the LUQ, or from the LUQ to itself. **(D)** Empirical interpretation of the Gamma plane and the quadrants. Along the shape axis, the distributions change from the shape *a* = 1 memoryless exponential to the Gaussian range, with skewed distributions with heavy tails in between. **(E)** The EMD is used to track the magnitude of the shift from each estimated PDF in windows at t and t + 1, while the direction is tracked by the quadrant landing. This curve represents the evolution of the stochastic process and serves to determine, e.g., critical points of transitions for each block of the session.

The Gamma was the best continuous family fitting the MMS in a MLE sense. The Gamma (a) shape and (b) scale parameters were then plotted on the Gamma parameter plane (Figures 3A,B). The Gamma family choice confirms previous work, as it has been found to be the optimal for representing MMS derived from human biorhythmic data registered from the face, eyes, whole body, heart, EEG, fMRI signals (e.g., Torres et al., 2013a; Ryu et al., 2021). This section is dedicated to explaining the empirical meaning of the Gamma parameter plane. We note here that at this level of analyses we are naïve as to the overall stochastic process and are empirically estimating its moment-by-moment evolution according to our unit of time (5 s window) chosen to yield tight confidence intervals.

The continuous Gamma family spans distributions of different shapes and different scales. Prior research has empirically characterized maturation of human neuromotor development, showing over the human lifespan a tightly linear relationship between the log shape and log scale of this family (Torres et al., 2013a; Ryu et al., 2021). As humans mature, distributions of the fluctuations in biorhythmic activities measured from their central and peripheral nervous systems grow more symmetric while the scale (dispersion) decreases. This characterization has reduced the parameters of interest to one (the shape or the scale) since knowing one, we can infer the other with high certainty. Focusing here then on the ranges of PDF shapes, we track the SL evolution. These parameters reflect different degrees of randomness and different levels of noise to signal ratio NSR (which in the Gamma family is given by the scale parameter of (equation 2).


(2) $$N\,S\,R=\frac{\Gamma \,\sigma }{\Gamma \,\mu }=\frac{a\cdot b^{2}}{a\cdot b}=b$$

We will use in our descriptions 1/NSR = SNR and will refer to it as the signal (empirically estimated mean over empirically estimated variance). Figure 3A shows the Gamma parameters estimated for each window in blocks 1 and 8, while Figure 3B shows the log-log Gamma parameter plane with a division into quadrants that reflect different empirical properties of the stochastic process. We take the median of the shape values and the median of the scale values and draw a line across each axis (Figure 3B), to break the Gamma parameter plane into quadrants that shift from window to window. Quadrants reflect the evolution of the stochastic process. Figure 3C shows the corresponding Gamma moments space following the color-code of Figure 3B whereby points that fall on the right lower quadrant (RLQ) are those representing symmetric distributions with low NSR (low dispersion), while those in the left upper quadrant (LUQ) represent distributions closer to the exponential range and having high NSR.

As an example, in Figure 3D, we summarize these results for empirical interpretation and inference in block 8. Generally, at the leftmost extreme, when the Gamma shape is 1, we have the special case of the memoryless exponential distribution (no points appear in this range for this example). This is the case of having a random process whereby events in the past do not inform more about events in the future than current events would. All future events are equally probable. The information is coming from the *here and now*. At this level of randomness, prior research has shown corresponding highest levels of NSR (We note that the signal to noise ratio SNR = 1/NSR will be used henceforth). Such distributions are typical to the motor code at the start of neurodevelopment (Torres et al., 2013a,^2016^). Around 4–5 years of age, when the system is (on average) mature enough to start schooling, receive instructions, and sustain longer attention spans, a transition into heavy tailed distributions is observed. By college age these distributions are tending to Gaussian, so that the shape parameter is at the other extreme of the shape axis on the Gamma parameter plane and the SNR is at its highest value (Figure 3D).

Prior work has also revealed that in systems where maturation is compromised (e.g., autism across the lifespan) these global signatures remain in the exponential range, randomly relying on the here and now and manifesting very low SNR. In this case, the system does not progress into acquiring a predictive code (Torres et al., 2013a).

For each Gamma PDF derived from the MMS in each window, the shape and scale parameters are plotted with 95% confidence intervals as points along a stochastic trajectory, on the Gamma parameter plane. Figure 3E makes use of the EMD to quantify the stochastic shifts from moment to moment in each learning block, as points transition from quadrant to quadrant.

### Dynamically tracking the stochastic signatures of the data

As the stochastic signatures *(a,b)* shift quadrants from moment to moment, they describe probability-positions over time (the dynamics of the stochastic process) on the Gamma parameter plane. Differentiation of this probabilistic positional trajectory yields an abstract velocity field whereby each velocity vector tangent to the trajectory, expresses the direction and the magnitude of the stochastic shift. To track the direction, we use the location of the landing point on the quadrants (the LUQ or the RLQ). The shift may leave the process in the same quadrant, or it may shift it away to the other quadrant. As shown in Figure 2D, to track the magnitude of the shift, we use the EMD scalar quantity representing the difference between the frequency histograms of amplitude fluctuations (MMS) derived from the EEG-hub channel activity. This is shown in Figure 3E for one participant’s activity in blocks 1 and 8. That is, the EMD value on the *y*-axis represents the difference between the histogram at time t and the histogram at time t + 1, taken at consecutive windows of activity. Notice that this is not physical distance. It is abstract distance in probability space. Likewise, this is not physical time, but time that depends on the length of the window and the overlapping % of the sliding window process.

### Global analyses

**FIGURE 4:**
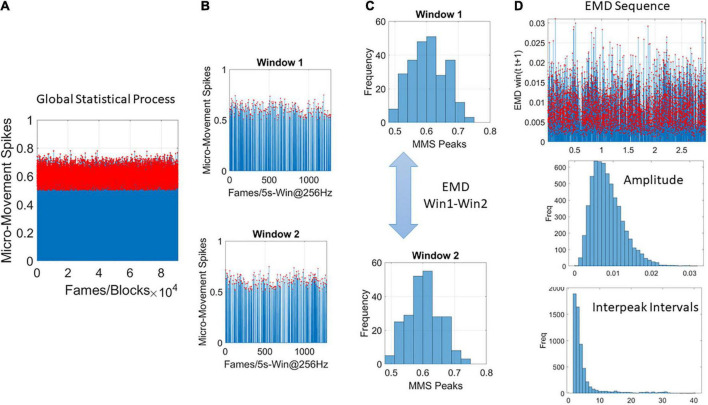
Global analyses **(A)** performed by pooling the MMS across trials and blocks and taking 5-s-long windows with 50% overlap **(B)** to obtain frequency histograms that can be compared using the EMD metric **(C)**. **(D)** Sweeping through the full trajectory of a condition gives the EMD sequence to obtain the peaks in red and gather them into a frequency histogram tracking the fluctuations in amplitude of the EMD variation (i.e., how the distribution change shape and dispersion) and the rate at which these changes occur as the inter peak interval intervals measuring the distances as well across peaks representing the PDF transitions. These histograms are used in MLE estimation of the distribution parameters best describing this global process.

As we accumulate the above discussed stochastic trajectories, we are locally tracking the shapes of the PDFs over the empirically estimated Gamma parameters. We use EMD to trace the moment-by-moment evolution of the stochastic Gamma process, as it unfolds across all trials and blocks. But initially we are naïve to the fluctuations in this process. It is then as we contemplate the full stochastic profile, *a posteriori*, that we can track the spikes of the EMD at a global time scale, i.e., across the entire session. This is shown in Figures 4A–D using the MMS and Gamma process once again, this time, the empirically estimation is on the fluctuations of the Gamma shape parameter representing the stochastic shifts of the distributions of the Gamma shape.

The general formula for the PDF of the Gamma distribution is shown below (equation 3), where *a* is the shape parameter and *b* is the scale parameter.


(3) $$f\;\left(x\right)=\frac{1}{\Gamma \;\left(a\right)\;b^{a}}\,x^{a-1}\,e^{-\frac{x}{b}}$$

Although the continuous Gamma family of PDFs can be parameterized with two parameters (*a* shape and *b* scale parameter), we can also obtain its statistical moments. We will use this alternative description of the distributions later to help visualize the results. The moments (μ, σ, skewness, kurtosis) are a⋅b,a⋅b2,2a,6k respectively.

### Results

### Behavioral results

The results from the analyses of the behavior and averaged potentials were previously reported (Vaskevich and Luria, 2018; Vaskevich et al., 2021). For completeness we summarized here the main behavioral result. Participants in the mixed group reached significantly slower reaction times than participants in both the correlated and random groups, even though the task contained a potentially beneficial regularity on half of the trials. This result replicated previous findings and highlights the crucial issue of validity: when the regularity is valid, applying this statistical information results in facilitation to both the search and response processes (correlated group). However, when the regularity is mixed with random trials, thus appearing within a relatively unreliable and unstable environment, a global interference effect emerges, so that the reliance on all prior information is attenuated. Previously proposed theoretical interpretation for these highly counterintuitive results were reported in Vaskevich and Luria (2018, 2019) and Vaskevich et al. (2021).

### Explicit memory test

In the mixed group, participants correctly classified previously seen layouts as familiar on 57% of the trials (hit rate), and incorrectly classified new layouts as familiar on 50% of the trials (false alarm rate). In the correlated group, participants correctly classified previously seen layouts as familiar on 55% of the trials (hit rate), and incorrectly classified new layouts as familiar on 48% of the trials (false alarm rate). For both the correlated and the mixed groups the differences between hit rate and false alarm were not significant, *F* < 1. The random group did not complete the explicit memory test as there was nothing to test for- there was no regularity in the task.

To assign a memory score (ET) we calculated the ratio between hit rate and false alarm rate for each participant. Higher scores correspond to better explicit memory of the visual layouts presented during the search task. The Overall memory scores of the correlated group (*M* = 1.37, *SD* = 0.9) and the mixed group (*M* = 1.25, *SD* = 0.7) were not significantly different, *F* < 1.

### Local level of the stochastic process

**FIGURE 5:**
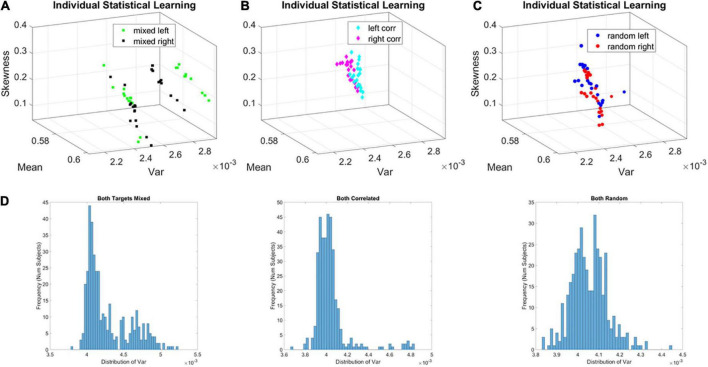
Local learning evolution captures two classes of learners in the unstable environment (i.e., mixed group). Empirically estimated Gamma moments span a parameter space whereby each participant represents a point by the moments of the probability distribution. The coordinates are the mean (*x-axis*), the variance (*y-axis*), the skewness (*z-axis*). The color represents the target orientation (left or right). **(A)** Mixed case (i.e., group) whereby trials intermix random and correlated conditions, spanning a relatively unstable learning environment. In this group two self-emerging distinct subgroups of participants. **(B)** Correlated group, for which layouts are consistent from trial to trial, spanning a stable learning environment. **(C)** Random group, for which layouts are generated randomly from trial to trial, spanning a stable learning environment where no regularity is present. **(D)** Corresponding frequency histograms of the distribution of the variance across trials, target types and participants.

For all three groups (correlated, random, mixed) we isolated the MMS from the continuous EEG data. We converted the fluctuations in the EEG amplitude (peaks μ*V*) and inter-peak-interval timing (*ms*) to unitless, standardized MMS trains that were then analyzed using a sliding window of 5 s with 50% overlap (see section Methods). The window-by-window analyses for each participant revealed two subgroups in the mixed group. On the Gamma moments parameter space, along the Gamma variance dimension, one subgroup of learners (subgroup A of *broad-variance learners*) expressed higher variance of the fluctuations in the MMS amplitudes at the start of the experiment. This departure from the other subgroup (B of *narrow-variance learners*) can be appreciated individually for each participant over the entire experiment in Figure 5.

**FIGURE 6:**
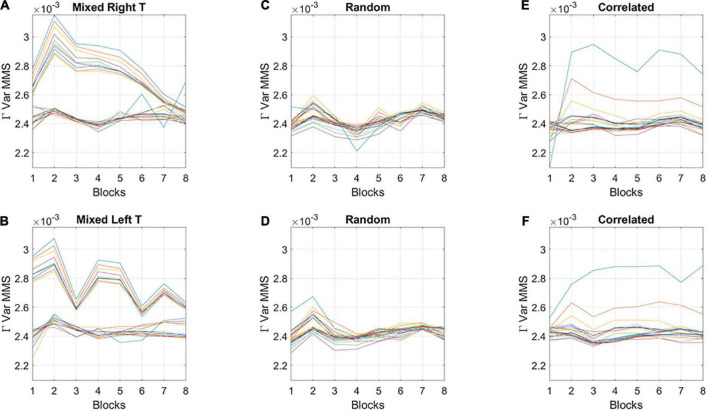
Broad- and narrow-variance groups according to the empirically estimated Gamma variance parameter block by block. **(A,B)** Two subgroups in the mixed group are revealed for right- and left-oriented targets (each curve represents the trajectory of a participant within the group). The subgroup with lower variance and narrower bandwidth of values throughout the experimental session separate from those in the subgroup with high variance and broader bandwidth of values. However, both subgroups converge to similar variance levels toward the 8th block of learning. Target types show different trajectories but similar convergence trend. **(C,D)** Random group shows similar levels of variance and stable learning throughout the experimental session, as does the correlated group **(E,F)** (with two outliers).

The fluctuations in the empirically estimated Gamma variance were then unfolded over blocks for each participant (Figures 6A,B). After the second block of trials, the levels of variance derived from the MMS-amplitude in subgroup A systematically decreased, eventually converging to the much lower level of the subgroup B. As such, subgroup A, with the initially much higher variance, expressed a higher bandwidth of overall variance values than subgroup B, which started out with much lower variance and remained in that regime throughout the eight blocks of the experiment. This was the case for both target types. Furthermore, this low range of variance in subgroup B was comparable to the ranges of variance observed in the random and correlated groups. This can be appreciated in Figures 6C,D for the random case and Figures 6E,F for the correlated case.

To show the overall differences in stochastic signatures of each case, we pooled the Gamma variance data from all blocks and for each mixed, correlated, and random group respectively (Figure 5D). The mixed group is indeed significantly non-unimodal, according to the Hartigan dip test of unimodality, *p* < 0.01 (Hartigan and Hartigan, 1985). The PDF derived from the MMS amplitude of the mixed group significantly differed from those in the random and correlated groups, according to the Kolmogorov Smirnov test for two empirical distributions (*p* < 0.01).

### Relationship between behavioral outcomes and stochastic results

**FIGURE 7:**
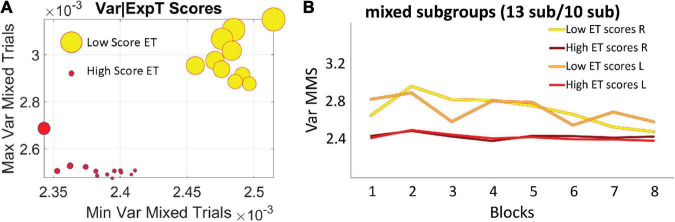
Self-emerging subgroups in the mixed group are differentiated by the scores of the explicit memory test. **(A)** The horizontal axis comprises the minimum value of the variance, while the vertical axis comprises the maximum value of the variance for each participant. Thus, the graph depicts the full range of variance values. The size of the marker is proportional to the explicit memory test score and the color represents the subgroup, with no overlapping between the two sets of participants. **(B)** Empirically estimated Gamma variance parameter unfolded block by block as in Figures 6A,B, for the two subgroups of the mixed condition. The group with less explicit knowledge [lower scores on the explicit memory test (ET score *M* = 0.94, *SD* = 0.4)] starts out with higher variance of the fluctuations of the MMS amplitudes (broad-variance group A), eventually converging to the much lower variance level of the subgroup that showed higher explicit knowledge of the regularity (ET score *M* = 1.52, *SD* = 0.75) (narrow-variance group B).

The two subgroups broad-variance A and narrow-variance B of the mixed group did not differ in reaction times or accuracy, suggesting that all participants were able to reach the same level of online performance. Instead, they were differentiated by their explicit knowledge of the regularity imbedded in the task, as reflected by their memory scores in the explicit memory test: 10 subjects in the broader variance subgroup A, *M* = 0.94, *SD* = 0.4 *vs*. 13 subjects in the narrow variance group subgroup B, *M* = 1.52, *SD* = 0.75, *p* < 0.01 non-parametric Wilcoxon ranksum test (Figure 7A). The subgroup A with broader bandwidth of variability showed low test scores, thus exhibiting less explicit knowledge of the regularity. In contrast, the subgroup B with the narrow, steady bandwidth of variability, gained a higher level of explicit knowledge, as reflected in higher explicit memory test scores (Figure 7B). We coined the process showing higher variance with low explicit memory score (subgroup A) “*exploratory mode.*” In contrast, we called the process showing lower variance and high explicit memory score “*error-correction mode*” (subgroup B). Here the mode refers to learning mode or phase and in the next results, we provide a stochastic characterization of these two fundamentally different modes of learning which, nevertheless, converged in block 8 to a similar variance range.

For completeness, the memory scores of the correlated group were also examined. Overall, memory scores (*M* = 1.37, *SD* = 0.9) were like the scores observed in subgroup B of the mixed group. This result is consistent with the similar stochastic learning signatures of the correlated group and this high memory subgroup (observed in the variance trajectories of Figure 6). We here infer that as the regularity in the correlated group was highly reliable, with layouts repeating on all trials, it seems that all participants reached some minimal level of explicit knowledge, therefore no subgroups emerged.

### Global a posteriori stochastic analyses of distribution shapes

Analyses of the stochastic signatures derived from pooling all trials, block by block, across all participants allowed us to examine the evolution of the distribution of the empirically estimated Gamma shape parameter, i.e., as the system experienced the learning and the PDFs shifted shape. The moment-by-moment fluctuations in the shape parameter provide insights into the dynamics of the stochastic process. Notice here that in our local computation (i.e., the MMS distributions at each window), we were naïve to the global dynamic nature of the stochastic Gamma process, as we were locally estimating the Gamma parameters (shape and scale) and the Gamma moments. Upon estimation of the full stochastic trajectory across the entire session, trial by trial and block by block, we are no longer naïve to the process. As such, we can make a global statement at the time scale of the entire session.

**FIGURE 8:**
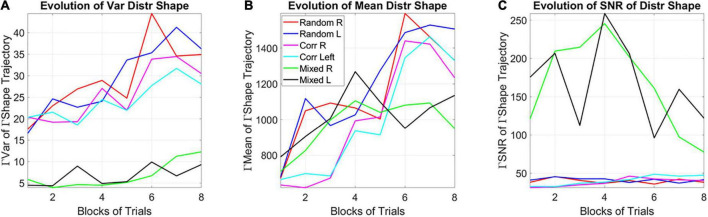
Learning evolution taken globally across participants and full session, shows the unstable environment (mixed group) to provide the most efficient conditions for learning, as indicated by the highest SNR. **(A)** Tracking, block by block, the empirically estimated variance of the distribution of gamma shape values obtained from the fluctuations in MMS amplitudes for each type of stimulus and target. Correlated and random groups trend upward with a steeper rate for correlated, while the mixed group stabilizes after ½ the session. The variance separates the correlated and random groups from the mixed group, with a marked reduction on the variability of distribution shapes and an overall trend to increase the variability in distribution shape toward the final blocks. **(B)** Tracking, block by block, the empirically estimated mean value of the distribution of shape values from the fluctuations in MMS amplitudes. **(C)** The signal to noise ratio (mean/variance) then shows the highest signal for the mixed trials, with a downward tendency after ½ the total session.

Among the moments of the distributions of the shape parameter, the variance of the evolving Gamma PDF shape parameter revealed the separation of the mixed group from the random and from the correlated groups (Figure 8A). Furthermore, a distinction is also observed for the mean parameter of the distribution of Gamma shapes (Figure 8B). As such, the SNR shows the highest signal content for the mixed group (Figure 8C). For both the correlated and random groups, the distribution shape has an increasing trend, consistent in both cases for the right- and left-oriented targets. However, in the mixed group, there is an initial increase in the shape that decreases and stabilizes by the 4th to 5th block, at much lower values of the variance, so that the SNR of the mixed group is much higher than that of the random or correlated groups. This elevated SNR indicates that the mixed environment is much more effective for learning than environments that contain purely random or purely correlated trials alone. Its information content is higher.

### Unfolding the gamma process for each learning mode

We show the stochastic shifts of each of the error correction (lower Gamma shape variance and higher explicit memory test score) and exploratory (higher Gamma shape variance and lower explicit memory test score), as they unfold across the blocks.

**FIGURE 9:**
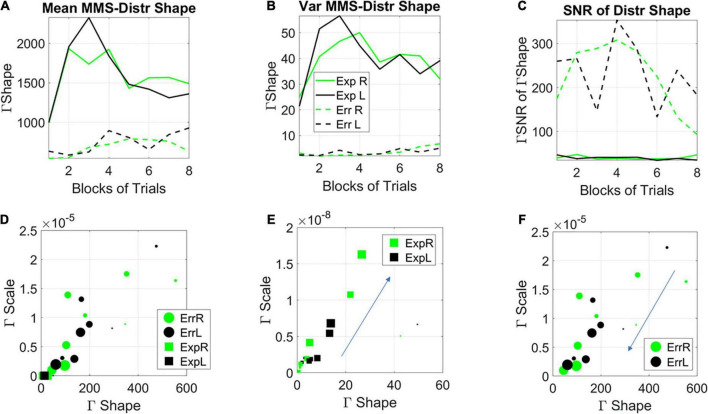
Stochastic characterization of exploratory *vs*. error correction modes across blocks by subgroups. **(A)** The evolution of the empirically estimated mean based on the distribution of Gamma shape values extracted from the MMS. **(B)** The evolution of the empirically estimated variance of the distribution of Gamma shape parameters. **(C)** The SNR (mean/var ratio) for the exploratory and error-correction subgroups. **(D)** Block by block evolution of the empirically estimated shape and scale parameters of the continuous Gamma family of probability distributions. Block number is proportional to the marker size, with earlier blocks having smaller size and later blocks increasing in size. The exploratory mode is confined to the gamma shapes close to the memoryless exponential distribution, while the error corrective mode evolves from higher to lower values of the Gaussian regime of the Gamma family. Unfolding each case [exploratory **(E)** and error corrective **(F)**] shows their convergence to a regime away from the memoryless exponential and tendency to more Gaussian like distributions. This convergent *global* behavior is congruent with the convergent *local* behavior of Figure 3.

The empirically estimated Gamma family shape parameters of the subgroup with high explicit memory scores (subgroup B) starts in the symmetric Gaussian range but trends down and converges towards the skewed, heavy tailed distributions, shown in Figure 9A for the mean Gamma shape and in Figure 9B for the variance Gamma shape of the two types of learners [the SNR (mean/var ratio) for the two subgroups is shown in Figure 9C]. The trajectory on the Gamma parameter plane (Figure 9D) confirms the departure from a memoryless random state (i.e., when the Gamma shape value is 1). To better visualize these processes, we zoom in and unfold the two types of learning modes of Figure 9D. Figure 9E focuses on the exploratory process. As time progresses, the learning generally evolves from memoryless (Gamma shape 1) towards skewed, heavy tailed distributions and more symmetric distributions of the shape. Figure 9F focuses on the error correction process. Here we see the opposite trend whereby initially the distributions have symmetric shape (in the Gaussian range of the Gamma family) but as time progresses, the distribution shapes approach values closer to those observed for the exploratory process: skewed, heavy tailed distributions.

Notice here that we are capturing the distribution of the fluctuations in the estimated Gamma shape parameter with a Gamma process as well. We are referring to the Gamma shape and Gamma scale parameters of the distributions derived (globally a posteriori) from the fluctuations in Gamma shape of the MMS derived from the EEG hub channels. On this Gamma parameter plane, the dispersion (Gamma scale of the fluctuations in Gamma shape value of MMS) along the *y*-axis, is larger as learning occurs, broadening the bandwidth of distribution shapes as learning takes place. The switch from exponential to heavy tailed to Gaussian distributions reflects the more systematic confirmation of a regularity in the stimuli. Initially, all future stimuli are equally probable (exponential regime), but in time, correct prediction of futures events increases, consistent with the transition from a detected regularity to a systematic goal. Once a goal is in place, error correction is the learning regime reflecting Gaussian predictive process embedded in this overall Gamma process. Here is where we see a tendency to symmetric shapes approached by both modes along the horizontal axis of the Gamma parameter plane. One mode (the exploratory) approaching it from the left, away from the memoryless exponential. The other approaching it from the right.

The stochastic transition depicted in Figures 9E,F confirms the separation between two fundamentally different learning styles with initially different stochastic regimes. It also highlights a phase transition approximately midway of the learning progression. Notwithstanding the initial differences, these regimes converged to similar signatures in the end. This transition from memoryless exploration (exponential) to predictive error-correction (heavy-tailed to Gaussian) surfaces in correspondence to midway of the session, blocks 3–4. Likely the regularity then self-emerges and eventually, through guess and systematic confirmation, transitions to a steady goal, one that serves to compute an error from.

In Figures 9E,F we see the system transitioning from an initial purposeless search to a search that then acquires a clear purpose, i.e., self-discovery of a task goal that was not instructed to the system. Our results suggest that this transition from memoryless into error correction-based learning depends on some minimum level of explicit knowledge. Examining this global process, we presume that in one subgroup enough explicit knowledge to trigger this transition was acquired much earlier than in the other subgroup. The group boasting an initial exploratory mode, for which the search was in the here and now, did not acquire distributions of the shape parameter away from the exponential range until around blocks 3–4. This was when the system shifted to a Gaussian mode (Figure 9E larger markers) and when locally the variance of the MMS shrunk (Figures 6A,B), thus spiking (globally) the SNR of the fluctuations in shape parameter (Figure 8C). In this exploratory scenario, the system does not immediately progress into acquiring a predictive code. In other words, because of not yet committing to regularities in the perceptual input, the predictive processing that underwrites exploitative or goal-directed behavior is initially precluded in favor of broadening the bandwidth of information that enables surprise and self-referencing towards the self-discovery of a goal. Only then, does the system transitions into an error-corrective regime.

### Dynamic statistical learning

At a global timescale (i.e., stochastic trajectory of the empirically estimated parameters examined *a posteriori*, across the entire experimental session) we assessed the change in stochastic variations of the signals over time. To do so, we examined the evolution of the fluctuations in the change of Gamma distributions’ shapes using the Earth Movers Distance (EMD) metric (see trajectories in the Supplementary Figures 2–4). We compared from trial to trial, and block to block, across participants, the fluctuations in the amplitude of the change in distributions of the Gamma shape parameter (as measured by the EMD). We also assessed the rate of the change in peaks (inter peak intervals related to the physical timing of the overall global process by our unit of time, 5-s windows with 50% overlap). These parameters are analogous to a kinematic “speed temporal profile” of the PDFs’ shape trajectory (Torres and Lande, 2015; Torres et al., 2016). As the Gamma process shifts stochastic signatures per unit time on the Gamma parameter plane, we obtain enough MMS peaks and estimate the Gamma parameter of each window with tight 95% CI. The EMD scalar profile over time, measuring how the histograms used in the estimation process change from window to window, reflect the dynamic nature of the stochastic shifts that occur as the participants perform the task and learn in exploratory, or in error correction mode, converging toward the signatures of the latter at the end of the learning process.

**FIGURE 10:**
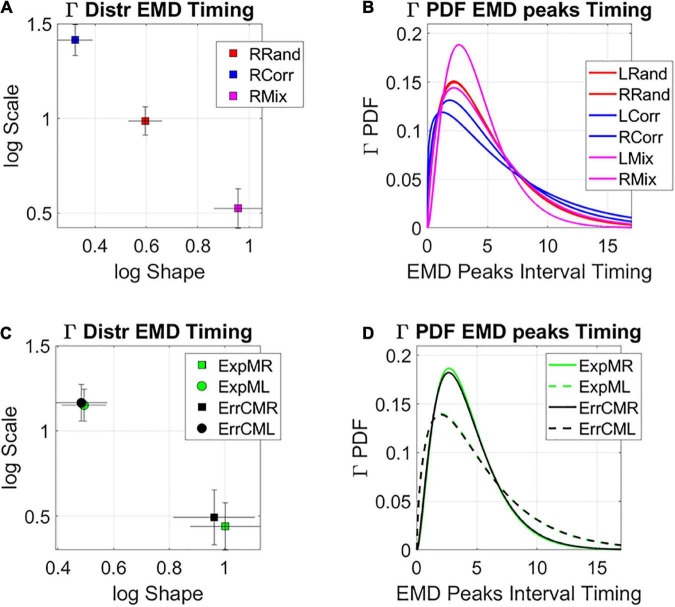
Global statistical learning dynamics. Unfolding the global rate of change in distribution shapes, as the system transitions from PDF to PDF, using the EMD to ascertain distribution differences from moment to moment. **(A)** Right target case is shown for the three groups with 95% confidence intervals for the empirically estimated Gamma shape and scale parameters. Each point represents a different distribution. Here the mixed group shows the maximal values of log shape (Gaussian) and SNR (1/log scale). **(B)** The PDFs corresponding to the maximum likelihood estimation (MLE) distributions in **(A)**. **(C)** Investigating the differentiation between targets for the two subgroups of the mixed condition at the global level reveals similar rate of change in the interpeak intervals, suggesting smooth transitions in both exploratory and error corrective cases. **(D)** Corresponding PDFs for **(C)**.

The analyses revealed that the system clearly distinguishes the rates at which the distributions change shape from the random to the correlated groups and between those and the mixed group. Figure 10A shows this on the log-log Gamma parameter plane where each point with 95% confidence intervals, represents the performance for the right target (left not shown for simplicity but has similar patterns, see Supplementary Figure 5). The corresponding PDFs for both right and left oriented targets are shown in Figure 10B. We can appreciate that the mixed case yields the most toward-Gaussian-predictive shifts in distribution change, with the highest shape value. This is accompanied by the highest SNR (i.e., at the lowest Gamma scale value). Furthermore, these rates of change in the two subgroups of the mixed case, clearly distinguish the left from the right oriented targets, with comparable rates of shifts in distribution shape for the exploratory and the error corrective subtypes. These are shown in Figure 10C (estimated Gamma parameters) and Figure 10D (corresponding Gamma PDFs). Different neural correlates of the learning process are shown in Supplementary Figure 6. These comparable shifts in distribution dynamics for exploratory and error correction stochastic regimes, hint at a smooth process whether the system is curiously wondering in exploratory mode, or aiming for a task goal, in error corrective mode.

## Discussion

This study evaluated online dynamics of SL using a new data type and analytical approach. This new platform relies on the moment-by-moment fluctuations in the signal of interest, which are traditionally discarded as gross data. Within the context of a visual search paradigm that manipulated, trial by trial, the reliability of stimulus regularities, while registering EEG signals, we examined the continuous stochastic process reflecting SL. We first isolated the EEG hub lead, maximally connected to other leads, and then proceeded to apply our new statistical analyses to this continuous data stream.

We found that SL is a highly dynamic and stochastic process, sensitive to the reliability of the incoming information. Moreover, we discovered that embedded in the gross data, traditionally discarded as superfluous noise under assumptions of normality, lies a code that describes different modes of learning. Based on our stochastic characterization of the learning phenomena at different local *vs.* global scales, we equate this distinction with two fundamentally different types of learning processes. These are the commonly studied error correction mode linked to stimulus regularity, and the newly characterized exploratory mode. This exploratory mode, stochastically characterized here for the first time, is likely reflecting surprising contextual variations that lead the system to eventually self-discover the purpose of the task with (i) the self-discovery of a goal through self-referencing and (ii) transitioning to the error-correction mode. Eventually the latter can lead to fast and accurate performance. To aid interpreting these results, we leverage prior research on the broad characterization of human biorhythmic activity (Torres et al., 2013a; Ryu et al., 2021) and reframe SL from the vantage point of neuromotor control, where spontaneous (seemingly purposeless) and deliberate (highly purposeful) motions coexist in any natural behavior from the start of life (Torres, 2011; Torres et al., 2016).

Two main results emerged from the current analyses. First, we show that unstable environmental conditions (i.e., mixing reliable and unreliable stimulus regularities) provide the most opportunity for learning, as characterized by higher SNR on both the global and local levels of analyses. Next, we show that on an individual basis, this unstable environment may give rise to different learning profiles: within this mixed group, two subgroups of participants self-emerged from the analyses. For one subgroup-B, coined error correction mode, the learning profile shows narrow variance in the MMS from start to finish and higher explicit memory test scores, reflecting better recall of the regularity. However, for the second subgroup-A, coined exploratory mode, the learning profile reflected an early stage of broad variance and memoryless learning which later converged into the signatures of the error correction mode. Crucially, this subgroup showed lower scores in the explicit memory test, as they did not recall the regularity with the degree of accuracy of the other subgroup. In their initial learning performance, all future events were equally probable, without a bias towards a particular regularity being reliably noted or recalled. We now turn to discussing each of these results in detail, while considering their implications on our understanding of SL in general.

### Unpredictable environments provide more opportunity for learning, corresponding to a more efficient learning process than predictable environments

When comparing the stochastic signature of learning within an unstable environment mixing the stimulus regularity between random and correlated trails (mixed group) with stable conditions providing reliable regularity (correlated and random groups), the process proved to be less stationary, more predictable in nature, and was characterized by higher SNR. These characteristics suggest that complex environments provide higher opportunity to learn than reliable environments. Moreover, within our theoretical framework, higher SNR corresponds to more efficient learning. These results are consistent with neuroimaging studies that have identified brain systems that track uncertainty in a curvilinear U-shaped function (Nastase et al., 2014; Hasson, 2017). Within these systems, full randomness or full regularity are alike in terms of informativeness and provide less information than the mixed case. As such, these systems seem to be especially sensitive to tracking relatively unreliable information in the environment.

Given that the real world is indeed complex, with our cognitive system continuously bombarded with variable regularities, it seems natural that we should be more attuned to learning under relatively unreliable (yet richer in information) conditions. However, suggesting that learning under such conditions is more efficient may seem to contradict the behavioral pattern previously observed in these data: participants in the mixed group reached slower RTs than both in the random and correlated groups (for a detailed account see Vaskevich et al., 2021). To resolve this issue, one must bear in mind that efficiency of learning is not necessarily manifested in online performance. That is, more complex learning conditions may hinder online reactions, but be beneficial for the long term. We propose that to gain further insight on SL, future studies should combine the methods introduced in the current work with experimental designs that involve changing regularities online and considering multiple sessions of learning. Indeed, such designs are becoming common within the field (Makovski and Jiang, 2010; Zellin et al., 2013; Vaskevich and Luria, 2019). However, so far, they lack the perspective of evaluating the dynamic and stochastic online evolution of the learning process, which is enabled by the methods used in the current work.

### Learning dynamics at multiple time scales

Within the SL domain, focusing on the dynamics of the learning process itself, with the specific consideration of multiple time scales, has been recently suggested as the next necessary step in SL research (Hasson, 2017; Frost et al., 2019; Conway, 2020). Experts in the field agree that to understand the neural substrates underlying behavior it is necessary to view it, and to measure it, as a continuous process, evaluating learning trajectories of its stochastic variations and learning stability. However, so far, this direction has not matured into meaningful research, largely due to limitations of the standard analytical techniques. To date, several measurements, such as rhythmic EEG entrainment (Batterink et al., 2019; Moser et al., 2021), functional connectivity (FC) analysis (Toth et al., 2017), and divergences in EEG activity in the beta-band (Bogaerts et al., 2020) have been used to assess the online signature of SL. Collectively, these studies show that during different tasks with embedded regularities the EEG signal changes over time to reflect SL. They provide insight into the mechanisms that are going through a transition during SL, such as task automaticity (Toth et al., 2017), and word representation (Batterink et al., 2019), thus complementing behavioral measures that rely on reaction times and accuracy. In the context of the present work, they provide solid justification for the choice of EEG recordings as the data used to assess the stochastic profile of SL. However, none of the previously proposed measurements are informative regarding the ongoing dynamics of the learning process itself, as in all the above-mentioned studies the signal is segmented into periods, with the relevant measurement averaged across many trials for each period, under the assumption of normality.

The present work goes beyond assumptions of normality, linearity and stationarity in the data and exploits the moment-by-moment fluctuations that prior work discards as gross data. Embedded in that gross data we uncovered the phase transitions in probability space that distinguished two fundamentally different modes of learning and revealed one (memoryless exponential) that converges to the other (predictive Gaussian). Both modes are well characterized by the continuous Gamma family of PDF s at the local level, when we are naïve to the upcoming moment-by-moment distribution, and at the global level, when *a posteriori*, we can see the fluctuations in the (Gamma) distribution shape unfolded through the Gamma process itself.

### Exploratory versus error correction modes differentiated by explicit knowledge of the embedded regularity

For a cohort of participants, the unstable environment (mixed group) triggered an initial stage of memoryless exploratory learning. During this stage, the stochastic signature of the process reflected a type of learning whereby initially all future events were equally probable. The stochastic signature unveiled in this initial period of learning for the broad-variance subgroup A of this cohort, suggests that the system was not relying on prior knowledge but was instead gathering as much information as possible from the “*here and now.*” Presumably, this exploratory stage was elicited by the high levels of surprise in an environment that contained rules that were not followed consistently over time. Crucially, this subgroup A also exhibited low scores in the explicit memory test. We posit that for participants in the narrow-variance subgroup B showing higher level of explicit knowledge, the stochastic signature reflected an error correction mode of learning throughout, from the beginning to the end of the task.

The behavioral differentiation between subgroups A and B, suggests that the transition from exploratory behavior into error correction depends on some minimal level of explicit knowledge that needs to be obtained. This conclusion contradicts the current assumption that both explicit and implicit SL always reflects error correction (Hasson, 2017; Frost et al., 2019). For instance, within theories arguing that both explicit and implicit learning systems operate simultaneously (i.e., dual-system approach), it has been suggested that during a learning episode, implicit associative learning occurs initially, which leads to the formulation of predictive “wagers” that steadily become more correct, leading to explicit awareness of the learned patterns (Dale et al., 2012). The initial stage of exploratory, memoryless sampling from the perceptual input that has emerged from our analyses has so far been overlooked.

The new methodology introduced in this work is grounded on deliberate *vs.* spontaneous movement classes (Torres, 2011), with different classes of temporal dynamics. Framed in this way, the error correction code would correspond to the deliberate movements intended to a goal. Such movements are well characterized by paths that can be traversed with different temporal dynamics and remain impervious to changes in speed (Atkeson and Hollerbach, 1985; Nishikawa et al., 1999; Torres and Zipser, 2004; Torres and Andersen, 2006). Within such learning, the path to the goal is independent of how long it takes to attain it and remains stable despite the moment-by-moment temporal structure of the stimuli, which must be learned and transformed into physical, motoric action (Torres and Zipser, 2002). This invariance is akin to timescale invariance in models of temporal learning, strongly supported by empirical data (Gallistel and Gibbon, 2000). In contrast, exploratory learning, would correspond to the class of spontaneous movements, i.e., highly sensitive to contextually driven variations in temporal dynamics of the stimuli (Torres, 2011; Brincker and Torres, 2018). These different dynamics can be distinguished in the variance profile of the learners in the mixed group of Figure 6A. They respond dynamically different across blocks, depending on target type. In this sense, exploratory trajectories with higher variance, lower explicit memory scores and fundamentally different target responses, are contextually more informative than error correcting trajectories. According to their initial exponential distribution signature, during this exploratory mode, all events are equally probable. The system samples without restriction. This mode may increase the chances of surprise, grabbing the system’s attention to some context-relevant events, perhaps self-discovering (through guess and confirmation of the regularity) the transition toward a consistent, ever more systematic state that may eventually result in a desirable, stable task-goal. At this point the system seems to enter and guide the error correction mode under a Gaussian regime. Such smooth transition across memoryless exponential, heavy tailed, skewed distributions to Gaussian modes are evident in the convergence of the variance profiles of the two subgroups in the mixed group to a common regime (locally obtained for the MMS Gamma variance in Figure 6A and globally computed in Figures 9E,F for the Gamma family of fluctuations in Gamma distribution shapes). Their smoothly evolving transition dynamics were also unveiled in the stochastic signatures of their rates of change (Figure 10).

We propose to trace back the newly characterized exploratory mode to the neonatal stages of learning. Such stages appear prior to the maturation of perceptual systems and are guided by endogenous bodily fluctuations that the infant senses from self-generated movements (likely heavily involving central pattern generators already operating at birth; Grillner and El Manira, 2020). To that end, we cite how neonates learn, perhaps supporting our idea that humans’ mental strategies and the different learning modes discovered here, are embodied, grounded on the types of learning that we ontogenetically transitioned through during early infancy, when seemingly purposeless movements preceded intentional ones (Thelen, 2001).

Studies of infants exploring an environment where the mother serves as an anchoring reference place, find that the babies explore using interleaving segments of progressive movements with lingering episodes (Frostig et al., 2020). They confirm that such exploratory behavior is homologous across species and situations (Drai et al., 2000; Frostig et al., 2020). Furthermore, a recent study from the SL domain demonstrated that infants prefer to attend to events that are neither highly unpredictable nor highly predictable (Kidd et al., 2012). The authors suggest that this effect is a characteristic of immature members of any species, that must be highly selective in sampling information from their environment to learn efficiently. We add to these interpretations a concrete stochastic model and suggest that infants attend to relatively unpredictable environments because these are ideal for the exploratory behavior that dominates early stages of surprise- and curiosity-driven motor learning in neonates (Torres et al., 2016) and infants (Torres et al., 2013a). Across early stages of life, when altricial mammals generally mature their somatic-sensory-motor systems (More and Donelan, 2018), human infants acquire a stable motor percept. As they undergo motor milestones (myelination, acquisition of motor, and sensory maps, etc.), the families of PDFs that are empirically estimated from their bodily biorhythmic motions, transition from spontaneously purposeless, memoryless exponential to intentionally purposeful, highly predictive Gaussian (Torres et al., 2013a).

Given our results, it appears that the exploratory type of learning is preserved throughout adulthood, and that there are conditions in which this exploratory, memoryless learning with high SNR, emerges on demand, and is likely extremely advantageous. An open question is, when is this type of learning beneficial? One possibility is that it supports flexibility within the system, as it provides it with a broader range of information that would have been missed by a premature systematic biasing toward a regularity, without allowing/evoking wondering behavior. That is, in changing, unstable environments, it may be best to initially gather as much information as possible, before committing to an error correction, goal-targeted mode. This direction, which is beyond the scope of the present work, may be tested by examining whether exploratory periods emerge during processes that require flexibly extending an existing solution to new context, known in motor control as transfer and generalization (Krakauer et al., 2006; Torres et al., 2013b; Wu and Smith, 2013; Tanaka and Sejnowski, 2015), but such studies are rare. This research may bear important implications for clinical programs that are currently grounded in animal models of conditional reinforcement that do not address the possible benefits of an exploratory mode of learning, whereby the value of a reward self-emerges internally from the self-discovery process, rather than externally given and *a priori* set by an external agent.

Related to these proposed processes, are recent models of human and machine learning that emphasize the role of curiosity within the learning system (Pathak et al., 2017; Dubey and Griffiths, 2020). These models suggest that the causal environment determines when curiosity is driven by novelty or by prediction errors. In an environment where the past and future occurrences of stimuli are independent of each other, the optimal solution for gaining a future reward is to explore novel stimuli. This novelty mode, that has been referred to as novelty-error-based (Dubey and Griffiths, 2020), and the standard prediction-error-based approaches have at their heart the same computational problem: optimize by minimization of an error that depends on a given targeted goal, while using prior information. Though also fueled in part by curiosity, the exploratory mode suggested in our present results is computationally different from the error correction mode. As explained, in our exploratory mode initially, all future events are equally probable, the SNR of the stochastic process is high, and the system does not yet operate with a goal in mind. In fact, it must self-discover it, gathering as much information as possible in a memoryless way, without yet committing to an objective function, a value function, a policy, or a reward. In this case, opposite to RL, Bayesian Reinforcement leaning and active inference, the system does not minimize surprise.

We argue that to characterize learning properly, this additional type of endogenous, curious *unexpected* exploration should be incorporated into future models of inference and learning. Indeed, intrinsic motivation and curiosity has become a dominant theme in machine learning and artificial intelligence over the past years (Daw et al., 2006; Baranes and Oudeyer, 2009; Schmidhuber, 2010; Still and Precup, 2012; Little and Sommer, 2013; Friston et al., 2017; Schwartenbeck et al., 2019). Perhaps the best example of this is active inference and learning (Friston et al., 2011, 2016). Active inference provides an account of optimal behavior in terms of maximizing the evidence for forward, world or generative models of engagement with the world. In other words, instead of learning to maximize reward, agents maximize model evidence or marginal likelihood (as scored with evidence bounds or variational free energy; Winn and Bishop, 2005).

In active inference, behaviors are chosen to maximize both expected value and expected information gain (i.e., expected free energy) (Parr and Friston, 2019). Statistically speaking, this ensures that behavior complies with both the principles of optimum Bayesian decision theory (Berger, 1993) and Bayesian design (MacKay, 2003; Parr and Friston, 2019). This leads naturally to an initial phase of exploratory behavior driven by expected information gain (a.k.a. expected Bayesian supplies, intrinsic value, epistemic affordance, etc.), which then gives way to exploitative behavior driven by expected value (a.k.a., prior preferences, extrinsic value pragmatic affordance, etc.). Our results speak of a different facet of this transition, namely one where *the system has no expectation whatsoever*. Instead, all future events are equally probable and signal information is at its highest, maximizing surprise. There is at this point, no gradient direction pointing the system towards descending error. During this initial naïve learning phase, the system casts a broad net over all incoming information that enhances the chance for a surprising event, before committing to any salience or regularity. This is precisely opposite to (complementary of) the minimization of predictive error or the consequences of predictive error. Crucially, the fact that the transition between the memoryless exploration mode and the error correction mode could be predicted from an independent assessment of behavioral data (i.e., explicit knowledge) lends a predictive validity to our analysis of the neuronal correlates of a new aspect of learning. Only after a goal self-emerges it can be incorporated into an objective function or model, transitioning from trial-and-error model-free, to error-correction model-based learning, as an objective function gets defined. At that stage, minimizing expected surprise, as in active inference, fits well with the error-correction phase that all participants eventually converged to. However, active inference, as other learning frameworks, will need to be modeled differently from its current conceptualizations of optimal expectation-driven exploration to include the newly discovered spontaneous and memoryless stage of learning.

Through the motor control lens, we posit that the new (expectation-free) exploratory mode described here, scaffolds the emergence of what we have coined *spontaneous autonomy* (Torres, 2018b), different from deliberate autonomy (i.e., derived from targeted error-correction). It will be critical to include random-memoryless, expectation-free exploratory learning with high signal content, in the future design of autonomous robots/agents. This type of autonomy can be realized through the self-referenced discovery of the relationships between actions and their consequences. The latter leads to the sense of action ownership and to the volitional control of physical acts that are congruent with one’s own mental intent (Torres et al., 2013b). We posit that only then, after acquiring this selectively adapted balance between autonomous and controlled acts, will others understand one’s intent and contribute, through co-adaptation, to the person’s agency.

We have in summary shown that using new analytical techniques, we can get a precise characterization of the dynamic nature of SL, the rich stochastic signal embedded in fluctuations that are traditionally treated as gross data and the differential nature of contrasting learning modes. Investigation is warranted on whether these results generalize to other SL paradigms, and to the acquisition of predictive information in learning in general. Of particular interest, are questions of individual differences, and the degree to which the exploratory and error correction learning modes may be differently recruited on demand by the same learner under different contexts. We here offer methods that allow to investigate these and many new questions in future SL research from the perspective of the nascent, developing motor systems and their richly layered dynamic and stochastic motor percepts.

## Data availability statement

The raw data supporting the conclusions of this article will be made available by the authors, without undue reservation.

## Ethics statement

The studies involving human participants were reviewed and approved by Institutional Review Board of Tel Aviv University. The patients/participants provided their written informed consent to participate in this study.

## Author contributions

AV ran the experiment, recorded the original EEG data, and analyzed the behavioral results. ET developed the novel analytical tools, analyzed the EEG data, and provided the statistical inference/interpretation from prior empirical and computational work. Both authors contributed to the conceptualization of this work, interpretation of the results, and the preparation of the manuscript. This work merges the motor control dynamic and stochastic perspective, brought in by ET, with the study of statistical learning, brought by AV.
