# Supplementary material for: Rethinking statistical learning as a continuous dynamic stochastic process, from the motor systems perspective
Source: Front Neurosci. 2022 Nov 8;16:1033776. doi: 10.3389/fnins.2022.1033776 (PMC9679382; doi:10.3389/fnins.2022.1033776)
Supplement: Supplementary file 1 [file Data_Sheet_1.docx]

Rethinking Statistical Learning as a Continuous Dynamic Stochastic Process, from The Motor Systems Perspective

Anna Vaskevich^1*^, Elizabeth B Torres^1,2,3,*^^‡^

^1^ Rutgers University Psychology Department, Sensory Motor Integration Lab

^2^ Rutgers Center for Cognitive Science

^3^ Rutgers Computer Science Center of Computational Biomedicine Imaging and Modelling

*Equal complementary contributions

^‡^ Corresponding Author

| 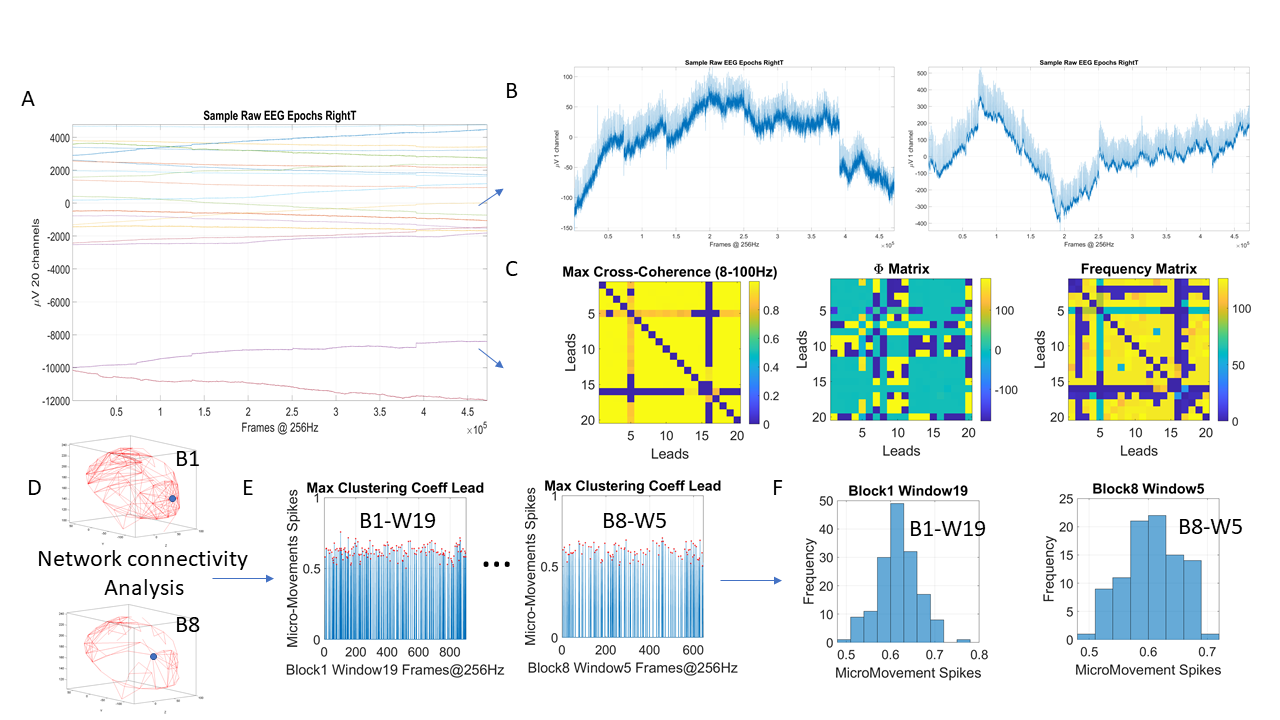 |
| --- |
| Supplementary Figure 1. **Determining the EEG hub lead**. Pipeline of network connectivity analyses to select hubs for stochastic analyses. (A) The electroencephalographic (EEG) activities from twenty channels and approximately ½ hour is sampled at 256Hz. (B) Two sample leads are used to instantiate the analyses. The pairwise cross-coherence is obtained. (C) For each pair, the maximal cross-coherence is obtained, with corresponding phase and frequency values at which the maximum is attained. These build three 20x20 matrices to parameterize (for each window and across each block of the session) the activity and build adjacency matrices (using the maximal cross-coherence matrix.) (D) The maximum clustering coefficient in each window is obtained, here represented in schematic form for Blocks 1 and 8 (using windows 19 and 5 for visualization purposes.) (E) The MMS are obtained, and the frequency histograms (as in Figure 2 of the main paper) used to obtain, pairwise, the Earth Movers’ Distance (EMD) matrix denoting how much effort it takes to convert one histogram into the other. This quantity allows us to track moment by moment (at 5 second window) how the stochastic signals best fitting the frequency histogram of MMS peaks change. For illustrative purposes we show MMS and histograms for block1 window 5 and block 8 window 19. |

Additional Results on Global Analyses of PDF transitions evaluated through the EMD (as explained in Figure 4 of the Methods section of the main text.

| 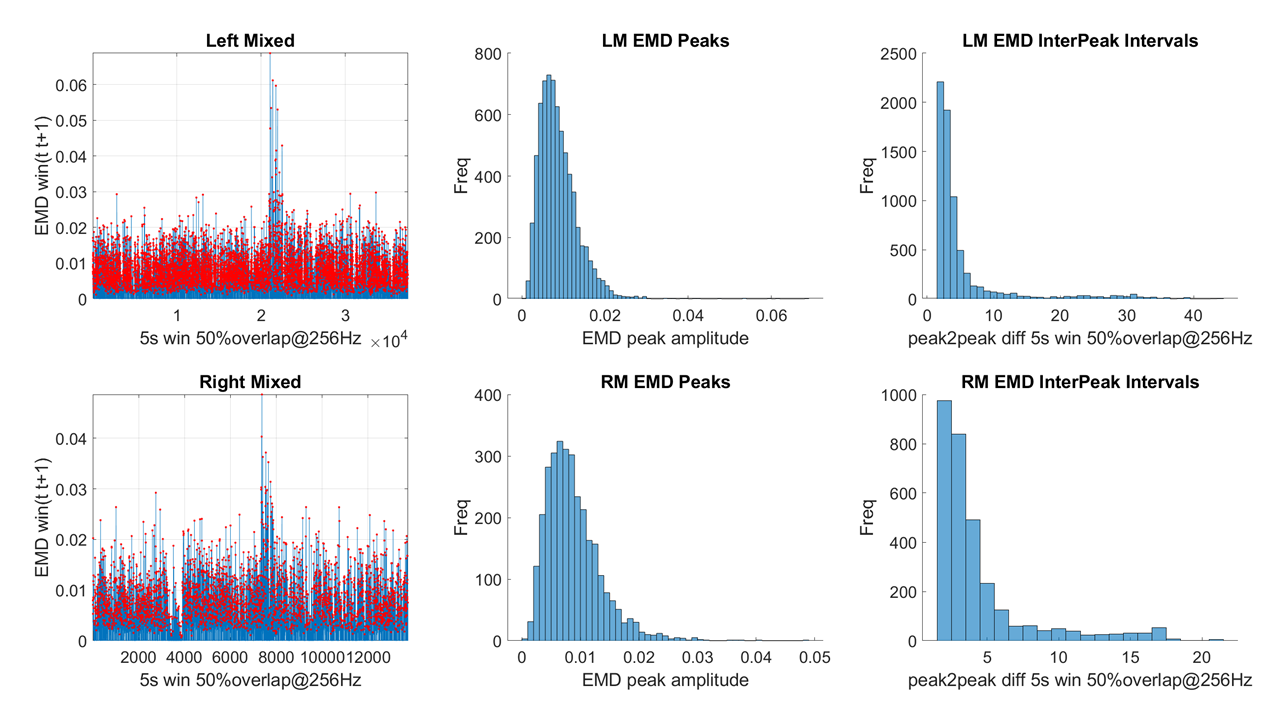 |
| --- |
| Supplementary Figure 2. Global analyses of the pooled data from all trials and blocks across participants for the mixed condition. Left and right oriented target trials on the top and bottom panels of the first column, respectively, showing the sequence of EMD values obtained by pooling across all data (order does not matter). Peaks (red) are used to build the frequency histograms depicted on the top (left target) and bottom (right target) panels of the second column. This are the fluctuations in EMD peak amplitude, quantifying the shifts in PDF empirically approximating the histograms using MLE. Panels on the third column show the frequency histograms of the interpeak intervals measuring the peaks’ distances from EMD window t and t+1, t represents the 5second-long window sweeping through the data with 50% overlap. |

| 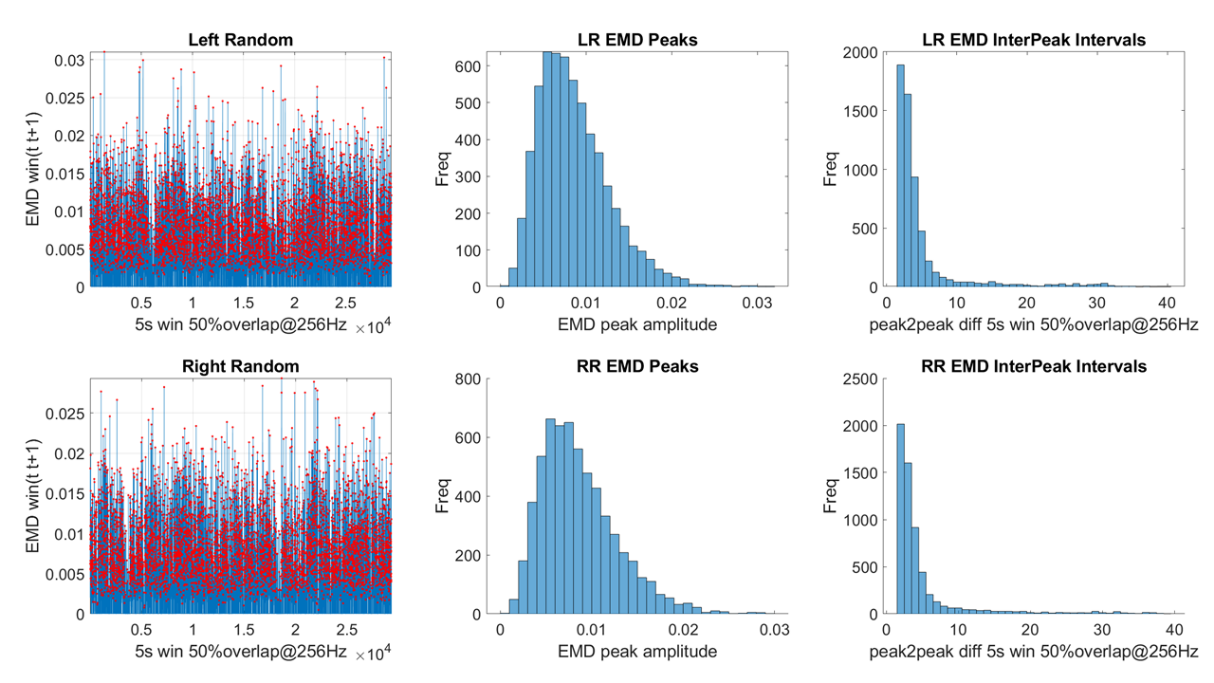 |
| --- |
| Supplementary Figure 3. Same as Supplementary Figure 1 for the random condition. |
| 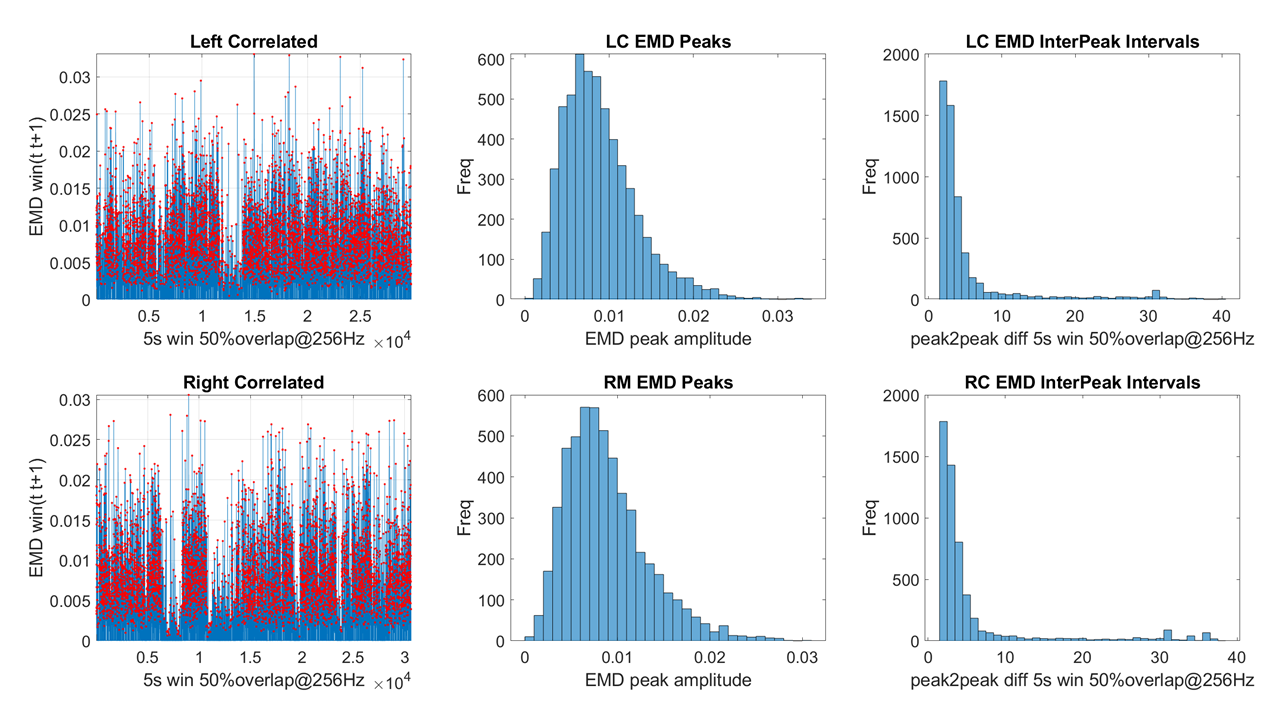 |
| Supplementary Figure 4. Same as Supplementary Figure 2 for the correlated condition. |

| 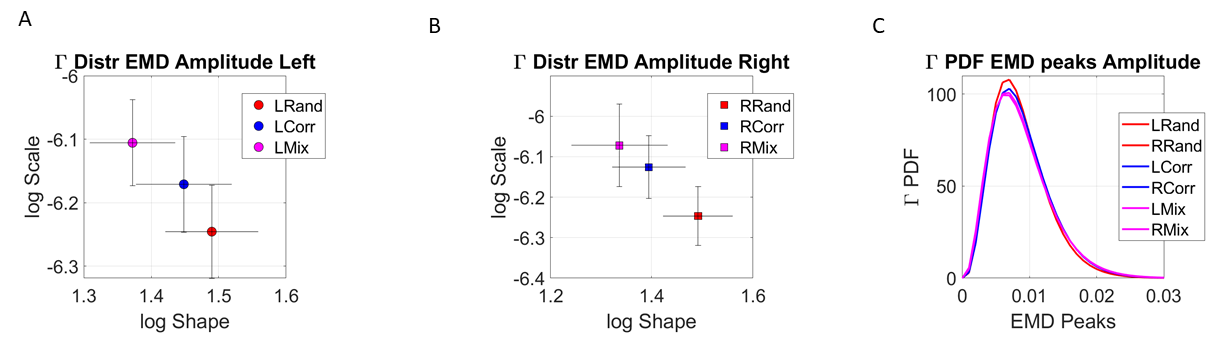 |
| --- |
| Supplementary Figure 5. Gamma process approximation of the global data for the fluctuations in amplitude. (A) Left oriented target global stochastic signatures mapped as empirically estimated parameters of the continuous Gamma family of probability distribution (see methods) plotted as points on the (shape, scale) parameter plane with 95% confidence intervals. (B) Same as in (A) for the right oriented target. (C) Corresponding PDFs estimated using MLE. |

| 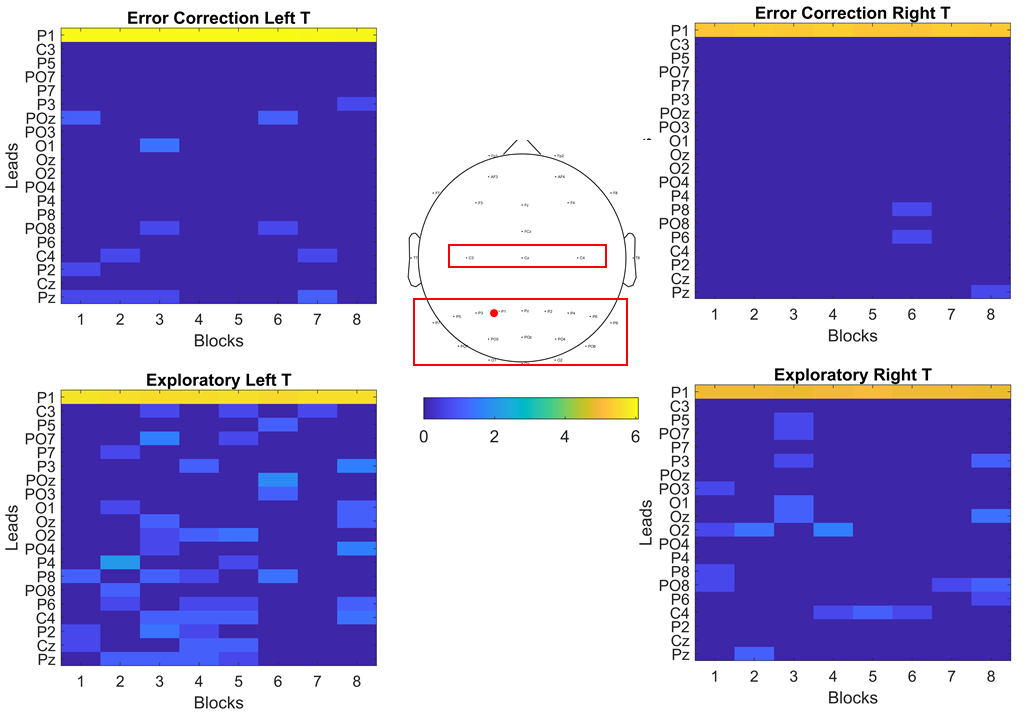 |
| --- |
| Supplementary Figure 6. Differentiation between error corrective and exploratory modes in the hubs’ activities across the leads under consideration. Center figure shows the leads used in the analyses. The leads acting as hubs of the network were quantified across trials and blocks (color bar in log scale) for each subgroup of participants, by pooling across participant type for all trials of each block in the mixed condition. The exploratory subgroup reveals a far more distributed code than the error corrective subgroup, with different patterns between target type. Notice the dynamic nature of this process as well, shifting from blocks 1 to 8. |
